# On the Effect Produced on the Circulation by the Long-continued Action of Cold Water Externally

**Published:** 1857-11

**Authors:** H. Bence Jones, W. Howship Dickinson


*On the Effect produced on the Circulation by the long-continued Action of Cold Water Externally. By Dr. H. Bence Jones and W. Howship Dickinson, Esq.*

1. The usual effect of a strong douche or shower-bath is the immediate depression of the pulse. By the first shock of water between 64° and 68° F. the pulse became weak and irregular, and may be reduced in rate even fifty beats in the minute. After the first shock the pulse recovers a little, but remains weak until the secondary effect or showering comes on, when it becomes weaker and intermitting, and may be quite imperceptible. After ten or fifteen minutes the pulse remains very small and weak, and shivering continues whilst the experiment lasts.

2. If the shower-bath is a small one (eight gallons), and the person taking it in good health, no great difference is perceived in the pulse, whether the water is hot (110°) or warm (74° F.)

If the water is very cold (47° F.) the pulse becomes smaller, but the rate is not affected.

With a shower-bath giving twenty gallons per minute a difference of twenty degrees (from 70° to 50° F.) causes a great difference in the shock. The difference in the after-effect, or shivering, is not so marked. The depression of the pulse when the shivering comes on is more continuous with the colder water, and is more manifest up to the end of the experiment.

3. When the pulse is raised above, or depressed below, its healthy standard, the shower-bath or douche produces very much less or a much greater effect than would be produced by the bath under ordinary circumstances.

As it seemed possible that a part of the reduction of the pulse might be due to the action of the cold water upon the capillaries and the radial artery in which the pulse was felt, a set of experiments were made, in which the forearm and hand were exposed to temperatures varying from 25° to 124° F. The results of these experiments may be thus stated:

1st. When one arm is in water at 50° and the other in air at 40° F., no difference in the pulse is observed in fifteen minutes.

2d. When one arm is in water at 110° and the other in air at 46° F., little, if any, difference could be felt in the same time.

3d. When one arm is in water at 44° and the other in water at 107° F., there was the same result in the same time.

4th. Even one arm at 33° and the other at 112° gave no result.

5th. Still lower and higher temperatures, 25° and 115° F., did not give any decided result in fifteen minutes.

6th. The douche-bath on the arm and hand, at 42°, produced no greater effect on the pulse than still water at 44° F.

Hence, generally, it follows that no part of the effect produced by the shower-bath upon the pulse depends on the action of the water on the hand and forearm in which the pulse is felt. —*Proceedings of Roy. Med. and Chir. Society, April* 14, 1857.

